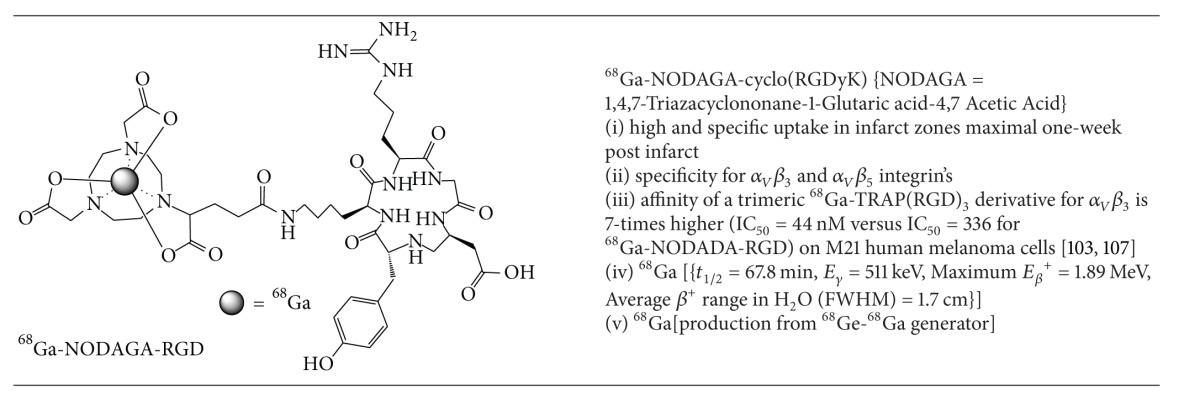# New SPECT and PET Radiopharmaceuticals for Imaging Cardiovascular Disease

**DOI:** 10.1155/2014/942960

**Published:** 2014-05-11

**Authors:** Oyebola O. Sogbein, Matthieu Pelletier-Galarneau, Thomas H. Schindler, Lihui Wei, R. Glenn Wells, Terrence D. Ruddy

**Affiliations:** ^1^Division of Nuclear Medicine, Department of Medicine, University of Ottawa, 501 Smyth Road, Ottawa, ON, Canada K1H 8L6; ^2^Division of Nuclear Medicine, Department of Radiology, Johns Hopkins University, Baltimore, MD 21287, USA; ^3^Division of Cardiology, Department of Medicine, University of Ottawa Heart Institute, 40 Ruskin Street, Ottawa, ON, Canada K1Y 4W7; ^4^Nordion Inc., 447 March Road, Ottawa, ON, Canada K2K 1X8; ^5^Canadian Molecular Imaging Center of Excellence (CMICE), University of Ottawa Heart Institute, 40 Ruskin Street, Ottawa, ON, Canada K1Y 4W7

## Abstract

Nuclear cardiology has experienced exponential growth within the past four decades with converging capacity to diagnose and influence management of a variety of cardiovascular diseases. Single photon emission computed tomography (SPECT) myocardial perfusion imaging (MPI) with technetium-99m radiotracers or thallium-201 has dominated the field; however new hardware and software designs that optimize image quality with reduced radiation exposure are fuelling a resurgence of interest at the preclinical and clinical levels to expand beyond MPI. Other imaging modalities including positron emission tomography (PET) and magnetic resonance imaging (MRI) continue to emerge as powerful players with an expanded capacity to diagnose a variety of cardiac conditions. At the forefront of this resurgence is the development of novel target vectors based on an enhanced understanding of the underlying pathophysiological process in the subcellular domain. Molecular imaging with novel radiopharmaceuticals engineered to target a specific subcellular process has the capacity to improve diagnostic accuracy and deliver enhanced prognostic information to alter management. This paper, while not comprehensive, will review the recent advancements in radiotracer development for SPECT and PET MPI, autonomic dysfunction, apoptosis, atherosclerotic plaques, metabolism, and viability. The relevant radiochemistry and preclinical and clinical development in addition to molecular imaging with emerging modalities such as cardiac MRI and PET-MR will be discussed.

## 1. Introduction: Planar, SPECT and PET Imaging, Radiotracers, and Molecular Imaging

The field of Nuclear Cardiology has rapidly expanded in the last four decades, which reflects an innovative and imaginative transition from subjective interpretations of planar images with less than ideal radiotracers to a digitally-based quantitative approach. Myocardial perfusion imaging has emerged as an increasingly valuable tool to identify and risk-stratify patients for subsequent intervention, medical management, or more interventive therapy with coronary angiography and possible revascularization. The rapid progression from planar imaging to single photon emission tomography (SPECT), positron emission tomography (PET) and magnetic resonance imaging (MRI) have been matched by an equally impressive progression and optimization of novel radiotracers that reflect the underlying molecular physiology of a variety of cardiac disease states.

These advancements provide improved diagnostic accuracy for disease detection and reduced effective radiation exposure while maintaining image quality [1–5]. New hardware designs such as dual modality systems (SPECT-CT, PET-CT, and PET-MR) and dedicated cardiac cameras with optimal detector geometric arrays, linear count statistics, and count rate response allow for lower-dose imaging, reduced scan time, and improved image quality [6, 7]. Novel software packages offer new reconstruction algorithms that include resolution recovery and noise suppression and so provide further increases in image quality [8].

One fundamental attribute that distinguishes nuclear imaging from CT and MR is the fact that a physiological and biochemical process is being imaged and not simply anatomy. Critical to the success of the past forty years was a drive to continuously understand the molecular processes underlying certain cardiac disease states and the development of novel radiopharmaceuticals that more suitably match this basic understanding. Molecular imaging offers the potential for targeted expansion of our understanding of the physiology that underscores various cardiovascular diseases beyond coronary artery disease (CAD) including inflammation, autonomic dysfunction, apoptosis, and angiogenesis. Combined advances in technology and radiopharmaceutical development have driven these changes prompting more powerful disease detection at earlier stages which portends earlier intervention, improved risk stratification, optimized diagnostic accuracy, and ultimately improved prognosis.

## 2. SPECT and PET Myocardial Perfusion Radiotracers for Detection of CAD

### 2.1. Current Technetium-99m Radiotracers Used for Myocardial Perfusion Imaging

**Figure 1:**
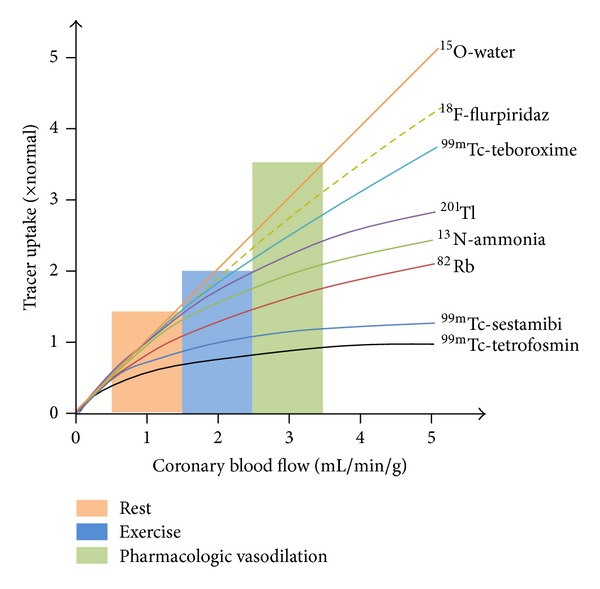
Schematic representation of cardiac PET and SPECT radiotracers uptake in relation to myocardial perfusion. ^15^O–H_2_O demonstrates close to linear uptake whereas the initial linear extraction of technetium-99m labeled compounds plateau at approximately 2 mL/min/g. PET radiotracers ^13^NH_3_^+^ and ^82^Rb^+^ fall between ^201^Tl^+^ and the ^99m^Tc-SPECT radiotracers, whereas ^99m^Tc-teboroxime demonstrates superior extraction at high flow rates. ^18^F-flurpiridaz rivals ^15^O–H_2_O with closer to linear extraction [11].

Viable myocardia are dependent on sufficient perfusion to deliver nutrients and oxygen to facilitate cell membrane and intracellular processes. This basic knowledge led to early infarction imaging with ^43^KCl as a marker of disrupted Na^+^/K^+^-ATPase activity [9]. Later radiotracers reflect changes in blood flow and myocardial extraction and this has become the basis for subsequent cardiac SPECT and later PET tracers for perfusion imaging. Thallium-201 (*t*_1/2_ = 73 hrs, *E*_X-ray_≅60–80 keV), first introduced in the early 1970s, has been extensively used as a perfusion radiotracer due to its favourable characteristics. This includes its high first pass extraction of approximately 85% and a better relationship between blood flow and myocardial uptake at higher flow rates during stress testing (Figure 1) [10]. Due to its differential washout between regions of high and low blood flow (redistribution) ^201^Tl is often used to assess myocardial viability in addition to ischemia. Although ^201^Tl has an excellent myocardial first pass extraction, its relatively low photon-energy renders myocardial perfusion images with ^201^Tl more prone to attenuation artifacts, in particular in the inferior wall, as compared to ^99m^Tc-labeled tracers with a 140 keV energy photopeak.

**Figure 2:**
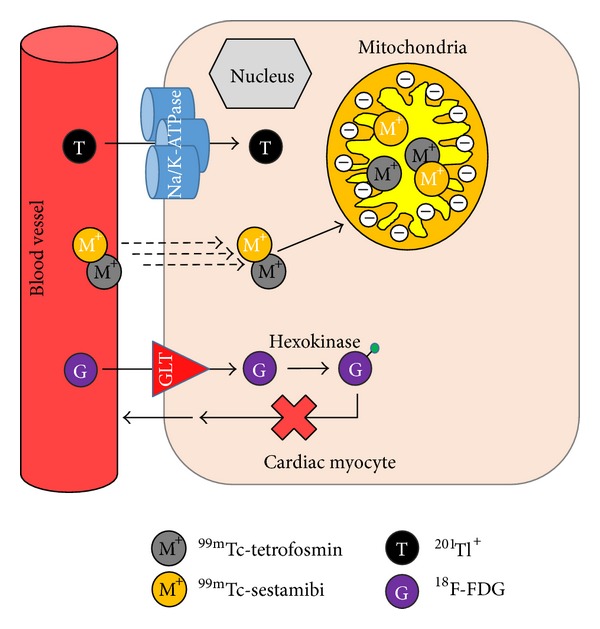
Schematic representation of the mechanism of uptake for commercial myocardial perfusion agents including ^201^Tl, ^99m^Tc-sestamibi, ^99m^Tc-tetrofosmin, and ^18^F-FDG (^18^F-fluorodeoxyglucose) in cardiac myocytes {GLT = glucose transporter; Na = sodium; K = potassium; green sphere indicates phosphate group}.

The ideal myocardial perfusion agent should have a linear relationship between myocardial uptake and blood flow, high first pass extraction over a wide range of blood flow rates, low extracardiac uptake, minimal myocardial redistribution, intrinsic chemical stability, and relatively straight forward radiochemical synthesis and purification. For SPECT, a family of technetium-99m (*t*_1/2_ = 6.02 hrs,  *E*_*γ*_ = 140 keV) based radiotracers was developed in the 1990's including ^99m^Tc-sestamibi (Cardiolite, Dupont) [12–14], ^99m^Tc-tetrofosmin (Myoview, GE Healthcare) [15], and ^99m^Tc-teboroxime (Cardiotec, Squibb Diagnostics) (Figure 2) [16]. Of these tracers, ^99m^Tc-sestamibi and ^99m^Tc-tetrofosmin have experienced widespread sustained clinical use and meet the majority of the aforementioned criteria (Table 1).

^99m^Tc-teboroxime, despite its initial FDA approval, is not in widespread clinical use. Due to the high initial uptake and rapid washout, image acquisition must occur within 2 minutes of injection, which is technically challenging. In earlier work, Beanlands et al. investigated ^99m^Tc-teboroxime in the context of postischemic injury and necrosis with low flow reperfusion [17]. Their rat model concluded that the uptake kinetics and clearance rates depend mainly on blood flow. Postischemic and necrotic tissue showed no significant variation in uptake. However there was a small but measureable reduction in clearance. This kinetic profile may represent a noninvasive approach to assess for inadequate reperfusion in acute myocardial infarction.

**Figure 3:**
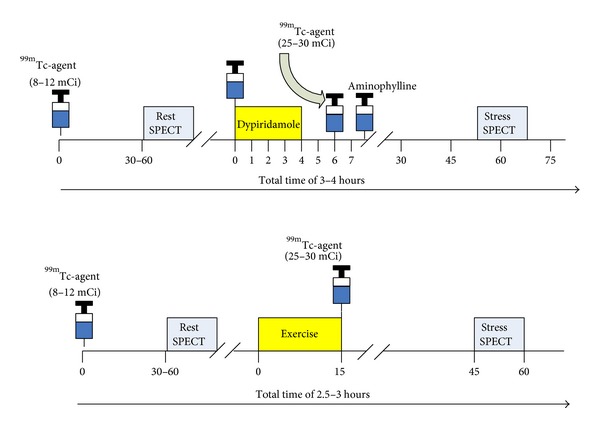
Single day rest/stress protocol with dipyridamole and exercise for myocardial perfusion imaging with SPECT at the University of Ottawa Heart Institute. Recently, radiotracer doses have been reduced by 50% with the use of new software and imaging technologies [3].

The diagnostic value of exercise and pharmacological SPECT myocardial perfusion imaging is well documented and previously reviewed [18]. The choice of exercise stress versus pharmacological stimulation with vasodilators (adenosine, dipyridamole, or regadenoson) or direct chronotropic/inotropic stimulation with dobutamine has well defined guidelines depending upon the goal of the assessment (diagnosis of obstructive CAD versus assessment of therapeutic response). Standard one day rest/stress protocols for pharmacologic and exercise stress is illustrated in Figure 3.

**Figure 4:**
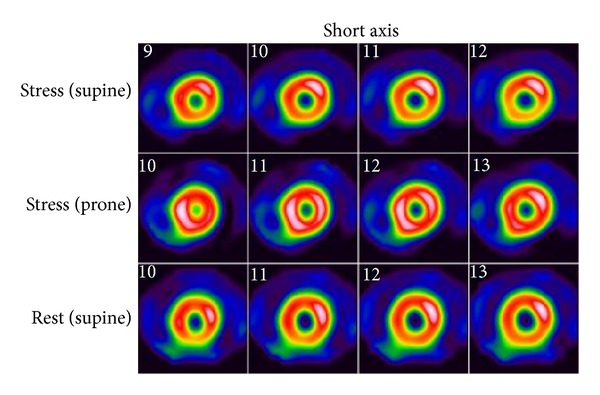
^99m^Tc-tetrofosmin rest/stress SPECT MPI study from the University of Ottawa Heart Institute in a 62-year-old female with delayed prone stress images (*middle panel*). Supine only rest/stress images suggest right coronary artery ischemia. Stress prone images show uniform radiotracer distribution confirming subdiaphragmatic attenuation artifact.

Myocardial perfusion imaging is indicated for the diagnosis of CAD, risk stratification, and evaluation of treatment response [19]. Although the accuracy of MPI has been well established, the pretest probability for CAD is an important consideration. In patients with low pretest probability of CAD, pooled data from 19 SPECT studies demonstrated a sensitivity ranging from 83% to 98% (mean of 92%) and a specificity ranging from 53% to 100% (mean of 77%) for ischemia detection indicating a high likelihood of false positive results [20]. For patients with intermediate or high pretest probability (e.g., significant risk factors, abnormal resting ECG, positive stress ECG, and typical or atypical chest pain), a positive result is likely to be true-positive and has a high negative predictive value [21, 22]. The specificity of MPI has improved with additional software and hardware (i.e., attenuation correction) [23] and patient manipulation such as prone imaging which can diminish artifacts including soft-tissue diaphragmatic attenuation or breast attenuation (Figure 4) [24].

Many clinical investigations have clearly validated the prognostic implications of a normal versus an abnormal SPECT MPI [25–27]. Derived from the accepted 17-segment model for regional evaluation of the left ventricle, semiquantitative assessments of perfusion abnormalities using indices such as the summed stress score (SSS) and summed difference score (SDS) have emerged as measures for describing the extent and severity of ischemia with predictive value for determining prognosis and functional recovery of systolic dysfunction [28].

A frequent indication for SPECT MPI in patients with suspected or known CAD is risk stratification. A normal SPECT MPI result warrants medical management or an alternative workup for a noncardiac cause. If reversible hypoperfusion is demonstrated on SPECT MPI indicating ischemia, these patients are often referred for invasive coronary angiography depending on the extent and severity of the defect and symptomatic status. Subsequently these patients may undergo revascularization with percutaneous coronary angiography (PCI) or coronary bypass surgery particularly if there is concordance between the hypoperfusion zone on MPI and angiography. The COURAGE (Clinical Outcomes Utilizing Revascularization and Aggressive Drug Evaluation) Nuclear Substudy with 314 of the 2287 total COURAGE patients compared the effectiveness of patients subjected to PCI + optimal medical therapy (OMT) versus OMT alone assessed with SPECT MPI [29]. The primary endpoint was a ≥5% reduction in ischemic myocardium on a follow-up scan obtained six to eighteen months after the initial study. Although the COURAGE trial showed no difference in long-term outcome, the nuclear substudy arm did demonstrate that patients in the PCI + OMT arm had a significant reduction in ischemia (33% versus 19%; *P* = 0.0004). This suggests that PCI + OMT reduces ischemia to a greater extent than OMT alone in CAD patients. However, the results should be viewed with caution since the subgroup was not randomized, and in those patients with moderate to severe baseline ischemia the differences were not significantly significant after Cox risk adjustment was performed. Thus Bourque and Beller suggest that “the COURAGE Nuclear substudy should be considered hypothesis generating rather than definitive” [28].

Despite widespread clinical use, ^99m^Tc-sestamibi and ^99m^Tc-tetrofosmin are imperfect radiotracers due partly to their nonlinear myocardial extraction at high flow rates (Figure 1) and high initial hepatic uptake, which can compromise interpretation of defects in the inferior wall due to adjacent photon scatter. Radiotracers that have minimal hepatic uptake or more rapid washout are continuously under investigation to ameliorate some of these limitations.

### 2.2. Novel Technetium-99m Radiotracers for SPECT Myocardial Perfusion Imaging

Bolzati et al. investigated a novel series of cationic ^99m^Tc-nitrido complexes containing a [^99m^Tc ≡ N]^2+^-core with a bidentate dithiocarbamate (DTC) and tridentate PNP-type bisphosphine chelates [30]. One example is the ^99m^Tc-nitrido complex DBODC5 (^99m^Tc-[bis (dimethoxypropylphosphinoethyl)-methoxyethylamine (PNP5)]-[bis (N-ethoxyethyl)-dithiocarbamate (DBODC)] nitride (N-PNP5-DBODC or N-DBODC5) (Table 2), which demonstrated high cardiac uptake and retention for more than 2 hours and nearly complete elimination of hepatocyte activity by two hours in Sprague-Dawley (SD) rats [31, 32]. The heart/liver ratio was 2 times better than ^99m^Tc-sestamibi at 30 minutes. Imaging studies demonstrated clear cardiac images as early as 15 min postinjection in SD rats for the ^99m^TcN-DBODC5 complex and rapid hepatic clearance. This complex is under clinical investigation as a novel MPI radiotracer [33].

Kim et al. have recently assessed the pharmacokinetics and biodistribution of ^99m^TcN-MPO ([^99m^TcN (MPO)(PNP5)]^+^: MPO = 2-mercaptopyridine oxide and PNP5 = * N*-ethoxyethyl-*N*,*N*-bis[2-(bis(3-methoxypropyl)phosphino)ethyl]amine)) in ten healthy subjects (Table 2) [34, 35]. After rest and stress injections (5 in each cohort) of 925 MBq of ^99m^TcN-MPO, whole body planar images were obtained. Hepatic uptake decreased over time from 20.88% at 10 minutes to 6.79% ID at 60 minutes. The 10 minute cardiac uptake was 2.47% ID at rest, which is comparable to ^99m^Tc-sestamibi or ^99m^Tc-tetrofosmin. At stress, the cardiac uptake was slightly higher at 2.57% ID and the heart/liver ratio was quite favourable at 0.27% ID, 60 minutes postinjection. The authors suggest that the rapid hepatic excretion is favourable for SPECT imaging particularly of the left ventricle.

Alternatively, complexes of the [^99m^Tc(CO)_3_]^1+^-core are particularly interesting since they are derived from the precursor [^99m^Tc(CO)_3_(OH_2_)_3_]^+^, which is an organometallic intermediate with three labile water molecules enabling coordination of bidentate or tridentate ligands [36]. This complex offers tremendous opportunity for diverse coordination chemistry and more importantly the ability to prepare targeted radiotracers. He et al. prepared a PNP (phosphorus-nitrogen-phosphorus) based bisphosphine complex containing the [^99m^Tc(CO)_3_]^1+^-core, [^99m^Tc(CO)_3_(15C5-PNP)]^+^. This complex also demonstrated high initial cardiac uptake, long cardiac retention, and rapid hepatic and pulmonary clearance [37]. Additionally the heart/liver ratio is 2.5-times that of ^99m^Tc-sestamibi.

More recently Goethals et al. have prepared the organometallic moderately lipophilic cationic derivative, ^99m^Tc(CO)_3_-tris(pyrazolyl)methane (^99m^Tc-TMEOP), and investigated the* in vitro* and* in vivo* biodistribution in Sprague-Dawley rats. Pharmacokinetic measurements in healthy male Wistar rats (*n* = 18) were compared with ^99m^Tc-sestamibi and ^99m^Tc-tetrofosmin (Table 2) [38]. Total cardiac uptake values of 1.28 ± 0.06 %IA (*P* < 0.05), 1.36 ± 0.11 %IA (*P* < 0.05), and 1.16 ± 0.05 %IA (%IA  =  percent injected activity) at 60 minutes were demonstrated for ^99m^Tc-TMEOP, ^99m^Tc-sestamibi, and ^99m^Tc-tetrofosmin, respectively (no statistically significant difference between ^99m^Tc-TMEOP and ^99m^Tc-sestamibi). Hepatic activity was also demonstrated to be statistically less in comparison to ^99m^Tc-tetrofosmin (*P* < 0.05 between 16.5 and 40.5 min) and ^99m^Tc-sestamibi (*P* < 0.05 from 16 min). Additionally significantly faster hepatic clearance was demonstrated. Based on the SPECT images the heart/liver ratio was over 2 times higher at 40 minutes for ^99m^Tc-TMEOP (6.98 ± 1.66; mean ± SD) compared with ^99m^Tc-tetrofosmin (2.66 ± 0.40) and ^99m^Tc-sestamibi (2.48 ± 0.30).

### 2.3. Novel Iodine-123 Radiotracer for SPECT Myocardial Perfusion Imaging

Mitochondrial complex-1 (MC-1) inhibitors show immense promise as molecular targets for novel cardiac radiotracers. MC-1 (also referred to as NADH: ubiquinone oxidoreductase) is the primary enzyme of the mitochondrial electron transport chain, which catalyzes the transfer of electrons from NADH to coenzyme Q10 (CoQ10). Due to the abundance of mitochondria in cardiac myocytes occupying nearly 30% of the intracellular volume, ^123^I, ^125^I, ^99m^Tc, and ^18^F based derivatives as inhibitors of the MC-1 complex have been investigated as targets for myocardial perfusion imaging. Structurally the MC-1 derivatives are analogues of known inhibitors such as rotenone, deguelin, piericidin A, and pyridaben [39–42].

Wei et al. have recently developed an iodine-123 labelled derivative of rotenone, denoted ^123^I-CMICE-013 (Table 3) [43]. In comparison to prior groups who investigated an oxidative destannylation route to iodination with tributyl-tin as the precursor, the Ruddy group developed an elegant one-step synthesis from a commercial rotenone precursor utilizing an iodogen oxidation approach across an alkene group that is peripherally located.

^123^I-CMICE-013 was isolated in greater than 95% radiochemical purity and high specific activity (14.8–111 GBq/*μ*mol; 400–3000 mCi/*μ*mol). MicroSPECT analysis in Sprague-Dawley rats demonstrated uniform myocardial biodistribution with sustained uptake and minimal extracardiac activity. In rats with left coronary artery ligation, absent perfusion was demonstrated. The two-hour postinjection heart uptake, heart/liver ratio, and heart/lung ratio was 2.01 ± 0.48% ID/g, 2.98 ± 0.93% ID/g, and 4.11 ± 1.04% ID/g, respectively.

**Figure 5:**
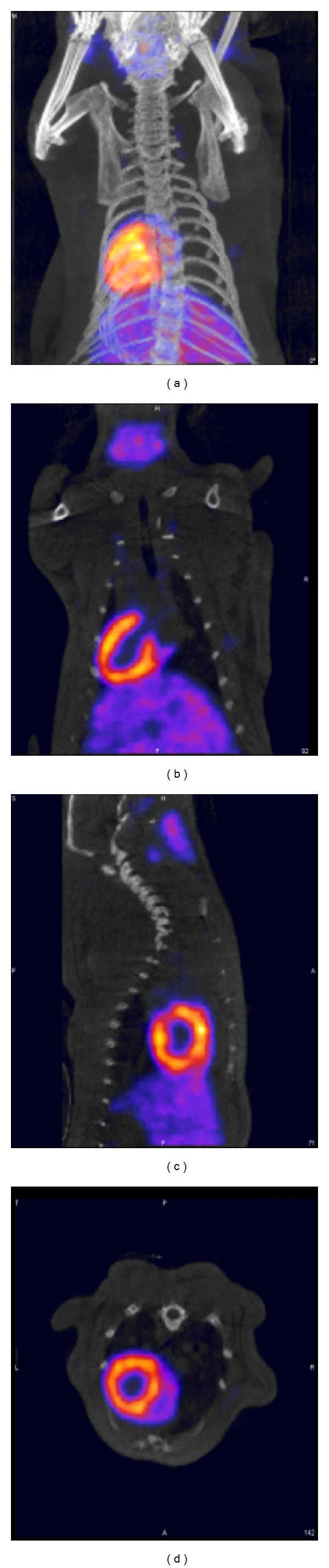
SPECT-CT images of ^123^I-CMICE-013 (mixture of isomers) 1-hour postinjection of 74 MBq in male Sprague-Dawley rats.

The oxidative iodination approach, however, produces several isomers requiring postprocessing steps to isolate the pharmacokinetically active substrates for optimal MPI. More specifically, the oxidative iodination of ^123^I-CMICE-013 produces four products that are two pairs of diastereomers, which are constitutional isomers of each other. Interestingly, the pharmacokinetics and biodistribution of the four isomers are complicated and not sufficiently explained by detailed structure-activity-relationship (SAR) theory. Accordingly, SAR predicts similar behaviour for the diastereomeric pairs with different biodistribution for the constitutional isomers. In fact, there appeared to be organ specific pharmacokinetic differences between the four isomers [43, 44]. For example, the diastereomeric pairs showed similar extracardiac uptake in the intestine, liver, and blood pool relative to one another; however, they demonstrated different cardiac uptake. The MicroSPECT heart/liver ratio correlated well with the* ex-vivo* biodistribution analysis for the first ^123^I-CMICE-013 diastereomeric pair (e.g., ^123^I-CMICE-013 A: 3.57 ± 1.47 MicroSPECT versus 6.63 ± 2.30 from biodistribution, *P* > 0.1;  ^123^I-CMICE-013 B: 1.89 ± 0.91 MicroSPECT versus 2.81 ± 1.45 from biodistribution, *P* > 0.1).  Figure 5 shows MicroSPECT/CT images of ^123^I-CMICE-013 in male Sprague-Dawley rats in three orthogonal projections at 1 hour postinjection. These results are promising for future clinical application.

### 2.4. Current Radiotracers Used for PET Myocardial Perfusion Imaging (^13^NH_3_, ^82^Rb^+^)

SPECT and its related cardiac radiotracers used for MPI dominate the field mainly for reasons of economics, availability, and portability of the ^99^Mo-^99m^Tc-generator to most nuclear medicine departments. However myocardial perfusion imaging with PET tracers has significantly improved diagnostic accuracy and prognostic ability compared to SPECT MPI [45]. Mc Ardle et al. recently demonstrated that in a direct comparison of ^99m^Tc-SPECT and ^82^Rb-PET MPI with ECG-gating and attenuation correction (excluding low-likelihood patients) the weighted-mean sensitivity and specificity were 90% and 91%, respectively, for ^82^Rb^+^-PET compared with 85% for both sensitivity and specificity for ^99m^Tc-SPECT [46]. Furthermore, the recent shortage of technetium-99m [47] and more favourable reimbursement rates from Medicaid/Medicare in the United States (no funding differential yet exists in Canada) for PET MPI support the routine use of this superior technology.

**Figure 6:**
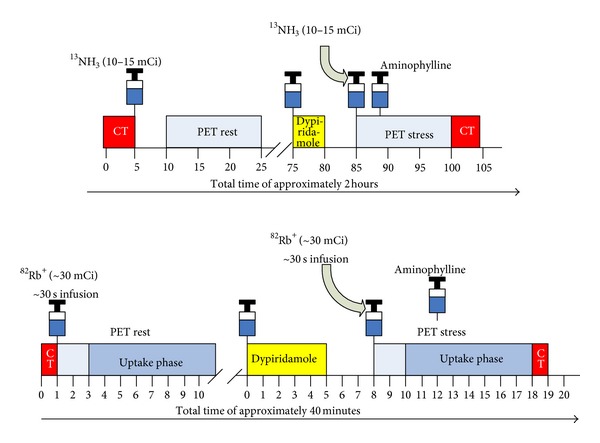
Typical rest/stress protocols for rubidium-82 and ^13^N-ammonia PET myocardial perfusion imaging at the University of Ottawa Heart Institute.

Initially investigated clinically in 1989, the availability of the ^82^Sr-^82^Rb generator for production of high specific activity ^82^RbCl obviates the need for an onsite cyclotron and continues to increase the relative volume of   ^82^Rb^+^ PET studies performed for cardiac MPI and functional assessments [48]. In comparison, ^13^NH_3_ does require an onsite cyclotron which limits its widespread utilization; however its physical properties and pharmacokinetics are superior to ^82^Rb^+^; thus ^13^NH_3_ continues to gain popularity. ^15^O-H_2_O has very limited use due to its very short half-life, poor contrast between blood pool and myocardium, and the necessity for an onsite cyclotron for production. Typical rest/stress protocols for ^13^NH_3_ and ^82^Rb^+^ MPI are illustrated in Figure 6. A rest/stress ^82^Rb^+^ PET MPI study is completed within an hour, which results in higher patient throughput and improved compliance. Given that each ^82^Sr-^82^Rb generator costs approximately $35,000, at least 4-5 patients per day are necessary for cost effectiveness [49].

There are multiple advantages of MPI with PET over SPECT. In addition to the improved diagnostic accuracy for detection of obstructive coronary artery disease, often equivocal SPECT studies (i.e., patients with large body habitus, muscular habitus with thick chest walls, or prominent breast attenuation) can be resolved with PET/CT due to integrated attenuation correction. The short half-lives are advantageous since higher injected doses can be administered for improved image quality while still permitting an overall reduction in effective radiation dose. PET has far superior sensitivity (100-times that of SPECT) and higher spatial resolution, particularly with ^13^NH_3_ and ^18^F-tracers given their respective positron ranges (Table 4). Moreover, the CT component of a PET/CT scanner can also be used to assess for coronary calcification, whereas the CT component of SPECT/CT systems are frequently not of diagnostic quality.

Another distinct advantage of PET MPI is the ability to obtain quantitative data of absolute myocardial blood flow which provides important corollary information beyond relative perfusion particularly in patients with multivessel disease. Absolute regional and global myocardial blood flow in mL/min/g can be determined at rest and following pharmacological stress. Typical rest flow values range from 0.5 mL/min/g to 1.0 mL/min/g and stress flow from 1.5 mL/min/g to 3.0 mL/min/g depending on the stress agent. Myocardial flow reserve (MFR), which is a ratio of rest and stress flow ranging from 2.0 to 4.0. MFR, has clinical utility in the detection of dypiridamole non-responders and subclinical microvascular dysfunction that may be missed with traditional relative perfusion imaging [50].

**Figure 7:**
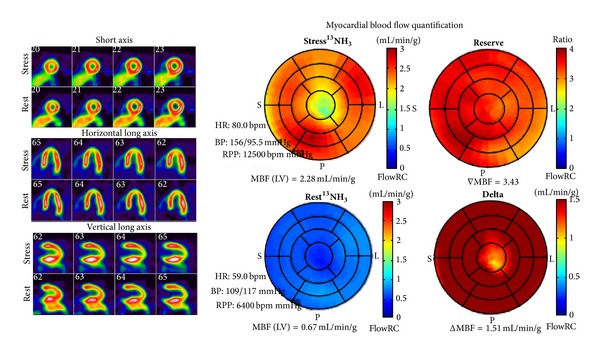
52-year-old female. Complaints of atypical chest pain. Risk factors include hypertension, dyslipidemia, and current smoker. ^13^NH_3_ PET MPI demonstrated normal myocardial perfusion with myocardial flow reserve (MFR)  =  3.34 {normal MBF >2.0). The combined normal MPI and MFR indicate an excellent prognosis.

Subclinical MFR or MBF abnormalities are presumed to represent endothelial or microvascular dysfunction prior to overt CAD. The prognostic utility of MBF and MFR has been shown to be an independent predictor of cardiac death in specific cohorts of patients (Figure 7). A normal MPI and MFR > 2 indicated an excellent prognosis with a “warranty” period of three years. In contrast, a normal MPI with MFR < 1.5 had a hazard-ratio of 2.4 for cardiac events. Ohira et al. suggest this cohort likely represents triple vessel disease or microvascular disease. Preventative therapy and possibly invasive coronary angiography are warranted [54].

### 2.5. New Fluorine-18 PET MPI Radiotracers: ^18^F-Flurpiridaz and ^18^F-FBnTP

^18^F-flurpiridaz (BMS-747158-02, Lantheus Medical, Inc.) is a novel PET mitochondrial complex-1 inhibitor indicated for detection of CAD and risk stratification (Table 5). Structurally it is an analogue of the known MC-1 inhibitor, pyridaben. The advantages of ^18^F-flurpiridaz over both ^13^NH_3_ and ^82^Rb^+^ are the nonnecessity for an onsite cyclotron and improved image quality due to the shorter positron range and longer half-life, enabling delayed imaging postinjection. The longer half-life of fluorine-18 provides an opportunity for exercise stress protocols, which are not possible with conventional PET MPI tracers. Moreover, the superior pharmacokinetic profile of ^18^F-flurpiridaz including its sustained myocardial extraction at high flow rates (Figure 1) enables the possibility of enhanced assessment of absolute quantification of myocardial flow reserve to better identify multivessel disease, microvascular disease, and response to treatment of endothelial dysfunction [11].

The intrinsic targeting capacity of ^18^F-flurpiridaz for mitochondria was demonstrated by Yalamanchili et al. who showed that initial high* in vitro* accumulation of the ^18^F-analogue in rat myocytes could be selectively inhibited by the administration of various inhibitors of MC-1 [55]. In a rat biodistribution model, ^18^F-flurpiridaz demonstrated very high uptake at 15 and 120 minutes postinjection with quite favourable heart/lung and heart/liver ratio at 60 minutes (12.7 ± 1.4 and 3.7 ± 0.2, resp.). As discussed, the pharmacokinetic profile of ^18^F-flurpiridaz is considerably improved over ^201^Tl^+^ and ^99m^Tc-sestamibi SPECT, which was confirmed by Yu et al. in a perfused isolated rabbit heart model [56]. They further showed that the net myocardial uptake and retention was significantly higher at all-time points for ^18^F-flurpiridaz compared to ^201^Tl^+^ or ^99m^Tc-sestamibi at physiologically relevant flow rates. Higuchi et al. showed that the heart/liver ratio of ^18^F-flurpiridaz was superior to ^13^NH_3_ in a rat model [41].

An alternative mitochondrial directed fluorine-18 labelled compound is F-18 fluorobenzyltriphenlphosphonium (^18^F-FBnTP)  (Table 11). Madar et al. showed rapid time-dependent accumulation of ^18^F-FBnTP in mongrel dog cardiac myocytes and prolonged retention [57]. Through MicroPET imaging the group was able to demonstrate that 68 ± 15% of the plateau activity was achieved within 15–30 seconds and sustained for 5 minutes postinjection.

In a comparative study of ^18^F-FBnTP and ^18^F-flurpiridaz in a rat model of short-term occlusion/reperfusion, ^18^F-FBnTP demonstrated prolonged lack of uptake in the defect area whereas over a similar period ^18^F-flurpiridaz showed slow restoration of uptake suggesting redistributive capacity analogous to ^201^Tl^+^. Thus, a rest/stress protocol with ^18^F-flurpiridaz with early and delayed imaging may be possible to investigate ischemia and myocardial viability [41].

Phase I clinical trials showed rapid renal clearance of ^18^F-flurpiridaz, high target/background ratios, overall favourable dosimetry, biodistribution, and safety and imaging characteristics after single rest injection [58]. The dose-critical organ at rest is the kidney (0.066 mSv/MBq) and the heart at stress (0.015 mSv/MBq with exercise and 0.019 mSv/MBq with adenosine). No adverse events occurred and a rest-stress dose of up to 14 mCi was validated.

The Phase II trial consisted of 143 patients from 21 centers who underwent rest-stress ^18^F-flurpiridaz MPI PET versus ^99m^Tc-sestamibi MPI with SPECT [59]. Eighty-six patients had confirmatory invasive coronary angiography. There was higher image quality (99.2 versus 88.5%, *P* < 0.1 for an excellent/good rating of PET versus SPECT MPI) and higher certainty of interpretation based on the percentage of cases with definitely normal/abnormal interpretation (90.8 versus 70.9%, *P* < 0.01 for PET versus SPECT MPI). In the 86 patients who underwent invasive coronary angiography (CAD defined as >50% stenosis),^18^F-flurpiridaz PET had superior sensitivity (78.8 versus 61.5%, resp., *P* = 0.02) and no appreciable difference in specificity (76.5% versus 73.5%, resp.).

The Phase II trial concluded successfully and the Phase III trial is ongoing since the fall of 2013 [60]. The primary objective is to assess the diagnostic accuracy of the detection of CAD with ^18^F-flurpiridaz PET MPI compared to SPECT MPI in single vessel CAD defined by invasive coronary angiography or a known and documented history of myocardial infarction.

## 3. Current SPECT and PET Tracers Used in Myocardial Metabolism and Viability

### 3.1. ^18^F-Fluorodeoxyglucose for PET Viability Imaging

The viability of myocardium after an ischemic insult reflects a continuum of metabolic derangement and dysfunction from stunned myocardium through hibernation to the process of remodelling. If the initial insult is not sustained and perfusion to the affected area is restored, then the myocardium will exhibit postischemic systolic dysfunction in proportion to the duration of the insult (stunning). Stunned myocardium is considered a transient dysfunction since there is complete reversibility. If there is repeated ischemic insult to the same area then the metabolic profile of the affected tissue is downregulated, stimulating intrinsic cellular protective mechanisms including cytoprotective gene transcription. This is referred to as hibernation or hibernating myocardium. Despite the more pronounced dysfunction, the potential for reversibility remains if adequate revascularization can be achieved. Often periinfarct or perihibernating remodelling occurs with concomitant myocyte dysfunction, which may or may not directly benefit from revascularization depending on the extent of the dysfunctional myocardium.

The most widely used radiopharmaceutical to assess viable myocardium is ^18^F-fluorodeoxyglucose (^18^F-FDG)  (Table 6). In the past, the redistributive property of thallium-201 was exploited since delayed uptake in a perceived infarct zone indicated viability. However the superior diagnostic quality of PET with ^18^F-FDG has replaced the use of ^201^Tl in most centers where PET is available.

Typical protocols for a complete viability study include an initial rest myocardial perfusion image with ^82^Rb^+^ or ^13^NH_3_. This is followed by the rest ^18^F-FDG acquisition where the patient first fasted for 6–12 hours and then received a glucose load (or hyperinsulinemic clamp). Normal myocardium demonstrates uptake of both the perfusion and metabolism radiotracer and scar demonstrates little to no uptake of either the metabolic or perfusion tracer (matched defect). Hibernating and thus viable myocardium is detected as having reduced perfusion and preserved metabolism (mismatched defect). Matched metabolism and perfusion defects are unlikely to recover following revascularization depending on the extent of left ventricular involvement.

**Figure 8:**
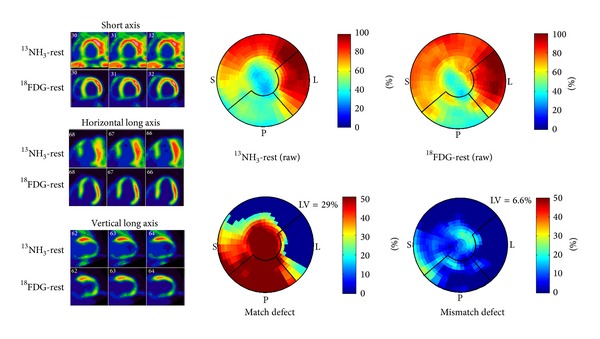
63M presented with NSTEMI. Coronary angiography demonstrated diffuse triple vessel disease (95% mid-LAD stenosis, 95% OM2 stenosis, 90% proximal LCx stenosis, 90% distal LCx stenosis, 95% OM1 stenosis, 95% ramus intermedius stenosis, 50–60% middistal RCA stenosis, and 60% right PDA stenosis). A ^13^NH_3_-^18^FDG PET viability study demonstrated approximately 29% of the left ventricle was scar with hibernating myocardium of 6.6%.

Multiple studies have demonstrated that if a small percentage of the left ventricle has evidence of hibernation (5–7%), there is a likely outcome benefit to revascularization [61]. Conversely, patients with large areas of matched metabolism and perfusion defects (scar) that are greater than 20% of the left ventricle are unlikely to have an outcome benefit to revascularization. Beanlands et al. validated this concept in a study of 70 patients with mean LVEF = 26% and quantitative separation of the extent of scar into tertiles (small, moderate, and large). They concluded an inverse proportionality between the extent of scar and the change in LVEF (9.0%, 3.7%, and 1.3% for small, moderate, or large scars, resp.) [62].  Figure 8 demonstrates a typical case from the University of Ottawa Heart Institute of a patient likely to benefit from revascularization based on the ^13^NH_3_-^18^F-FDG viability image interpretation, despite the large area of scar.

The role of ^18^F-FDG PET-CT has been expanded more recently to assess cardiac myocyte metabolism after cardiac resynchronization therapy (CRT).^18^F-FDG PET uptake has been shown to correlate with improved long-term left ventricular systolic function and ventricular remodelling [63]. Additionally, patients with severely symptomatic left bundle branch block or dilated cardiomyopathy who receive CRT have been shown to augment left ventricular metabolism which becomes more uniform across the ventricular myocardium. These changes restore the observed reverse mismatch where there is low ^18^F-FDG uptake-to-flow ratio particularly at the septum. This reverse mismatch has been used to predict those who may respond to CRT since up to one-third are nonresponders. Inoue et al. have demonstrated high accuracy for this approach in predicting response to CRT (ROC analysis AUC = 0.93) compared to measurements of LVEF (AUC  =  0.66) or QRS duration (AUC = 0.75) [64].

### 3.2. ^123^I-BMIPP (*β*-Methyl-Iodophenylpentadecanoic acid) Free Fatty Acid

^123^I-BMIPP is an iodinated branch chain fatty acid, first introduced by Knapp Jr et al. [65] (Table 7). Branch chain fatty acids are the predominant metabolic fuel for cardiac myocytes under rest conditions when metabolic demands are low and there is sufficient oxygen-rich blood for metabolism. BMIPP possesses a methyl substituent at the *β*-position of the fatty acid that alters metabolism in comparison to normal free fatty acids. Fujibayashi et al. demonstrated that BMIPP metabolism at rest not only depends on regional oxygen-rich perfusion but also on myocardial carbohydrate utilization [66]. This combination of the overall dependence of BMIPP pharmacokinetics on multiple factors results in a delicate and complicated balance of myocardial uptake, retention, and metabolism. In a canine model the Fujibayashi group showed that BMIPP demonstrates rapid myocardial extraction (74% of ID), high myocardial retention (65.3%), and low washout (8.7%) thirty minutes after injection [66]. However, these factors depend on whether or not *α*- and *β*-oxidation metabolites were present, which depends on myocardial carbohydrate metabolism. Overall, the oxidative degradation products are known to back-diffuse out of the cardiac myocytes which may complicate image interpretation in myocardial perfusion imaging. However, the time frame of back-diffusion is slow relative to typical SPECT MPI protocols. Despite the complicated metabolism, clinicians have experimented with BMIPP in several cardiac diseases including acute infarction.

#### 3.2.1. ^123^I-BMIPP SPECT in Acute Rest Myocardial Perfusion Imaging (ARMPI)

Acute rest myocardial perfusion imaging (ARMPI) has been shown to provide information about relative abnormalities of coronary blood flow before several of the currently utilized ischemic markers are detectable including cardiac biomarkers (CK, TnT, and TnI), ECG changes, or ventricular dysfunction. In the United States, 6% of evolving myocardial infarcts are missed and patients inappropriately discharged resulting in a one-month mortality of approximately 33% [67]. The roughly 50% percent of patients admitted with symptoms suggestive of ACS, 10–17% are confirmed indicating a high unnecessary admission rate at significant costs (>$5 billion) [68]. ARMPI was first introduced in the 1970's with ^201^Tl and later revisited with ^99m^Tc-sestamibi and ^99m^Tc-tetrofosmin in multicenter randomized controlled trials (ERASE Chest Pain - Emergency Room Assessment of ^99m^Tc-Sestamibi for Evaluation of Chest Pain) [69, 70].

The ERASE Chest Pain trial demonstrated improved triage decision making in symptomatic ED patients with diabetes when evaluated via ARMPI and the PREMIER trial validated the reliability of ARMPI for ruling out MI when applied to patients in developing countries. Udelson et al. performed ARMPI in a cohort of emergency patients presenting with chest pain and dyspnea with normal or nondiagnostic ECGs. They demonstrated a 10% absolute reduction and 20% relative reduction in rates of unnecessary admissions. Bilodeau et al. performed ROC analysis on their cohort of ARMPI studies stratified by symptom of pain and the location, which showed very high overall accuracy [67].

The limitations of the ARMPI study includes the inability to discriminate between coronary spasm and true ischemia from a fixed coronary stenosis. This reduced overall diagnostic accuracy. Additionally, the images in coronary spasm can be normal or even supernormal second to reactive hyperemia. Furthermore, the imaging study must be performed within 3 hours of symptom cessation since administration of rest radiotracer after this cut-off may significantly underestimate the extent of at risk myocardium and limit prognostic ability [71]. The lowest detection limit of ARMPI is roughly 3–5% of the myocardium. Below this value, ischemia and/or scar may be missed. BMIPP imaging offers a simple and effective screen for patients with suspected ACS to risk stratify those requiring admission and appropriate therapy and may be more accurate than ^99m^Tc-sestamibi or ^99m^Tc-tetrofosmin ARMPI.

Under rest conditions BMIPP uptake is essentially uniform across the myocardium with a preserved blood supply. However, under ischemic conditions with diminished availability of oxygen-rich blood, metabolism shifts to the glycolytic pathway where glucose utilization dominates. If perfusion is sufficiently restored in a timely fashion, the affected myocytes continue to utilize the glycolytic pathway, and thus there is a lag period before full restoration of free fatty acid utilization occurs. This lag period is referred to as “ischemic memory.” In the emergency setting of acute chest pain and/or ACS, BMIPP imaging is advantageous since images demonstrating reduced uptake reflect “ischemic memory” which can be detected as late as 48 hours after event, well beyond the period of coronary spasm. Additionally, BMIPP imaging is acquired at rest only which is advantageous in cases of relative or absolute contraindications to exercise or pharmacologic stress.

Kawai et al. investigated 111 patients presenting to the emergency department with acute chest pain and suspected ACS and all subsequently underwent catheterization [72]. ^123^I-BMIPP SPECT imaging performed within 48 hours of presentation demonstrated a sensitivity and specificity of 74% and 92%, respectively, for diagnosis of obstructive coronary artery disease. In a direct comparison of 87 patients imaged with ^99m^Tc-tetrofosmin on admission day and ^123^I-BMIPP on the subsequent day with coronary artery disease or spasm, ^123^I-BMIPP was able to identify 64 patients with abnormalities versus only 33 patients imaged with ^99m^Tc-tetrofosmin (*P* < 0.05). Moreover, BMIPP imaging demonstrated a higher severity score compared with ^99m^Tc-SPECT and had incremental prognostic implications as demonstrated by Kontos et al. in a multicenter study performed on 507 patients [73].

#### 3.2.2. ^123^I-BMIPP SPECT in Chronic Kidney Disease

There is a higher prevalence of coronary artery disease in chronic renal failure (CRF) patients, particularly on hemodialysis [74, 75]. Mitigated by insulin resistance, endothelial dysfunction particularly of smaller arterioles has been shown to play a role in the pathogenesis of CAD in CRF patients [76]. Conventional contrast agents are contraindicated, which precludes CT angiography. Other patients may not tolerate the exercise or pharmacological stress of traditional MPI; thus rest BMIPP is a viable alternative for detection of ischemia in this patient population.

Nishimura et al. first applied BMIPP imaging for the detection of ischemia in hemodialysis patients [77]. Their results demonstrated concordant findings in the left ventricle for ^123^I-BMIPP SPECT and coronary angiography. Furthermore, in a prospective assessment of 375 asymptomatic hemodialysis patients imaged with ^123^I-BMIPP versus ^201^Tl SPECT for 3.6 ± 1.0 years, the survival analysis showed that cardiac death-free survival rates at 3 years were 61% in patients imaged with ^123^I-BMIPP SPECT (summed score ≥12) versus 98% (summed score <12). This indicates that hemodialysis patients with severely impaired myocardial fatty acid metabolism are at high risk for subsequent cardiac events.

## 4. PET Tracers for Imaging Atherosclerotic Plaques

### 4.1. ^18^F-Fluorodeoxyglucose for Imaging Inflammation in Atherosclerosis

Atherosclerosis is a chronic arterial inflammatory process consisting partly of intimal vascular damage with remodelling. Imminent rupture is an active process with dynamic recruitment of inflammatory cells and markers consistent with inflammation, apoptosis, and angiogenesis. The understanding of the dynamic nature of these processes underpins the development of novel radiotracers for imaging purposes [78]. Recent investigations with conventional PET tracers such as ^18^F-FDG for imaging inflammation have proven more promising than earlier attempts at apoptosis imaging via ^99m^Tc-annexin-V with SPECT [79].

^18^F-FDG is a known inflammatory marker which accumulates in actively recruited and metabolically active leukocytes, specifically macrophages. To adequately image the inflammatory process of coronary atherosclerosis, the endogenous myocardial uptake must be adequately suppressed. This is typically achieved by conventional fasting protocols where the patient restricts all food intake and is maintained essentially on clear fluids for 6–12 hours prior to injection of FDG.

Early preclinical investigations in rabbit models of iliac artery atherosclerosis showed congruent macrophage recruitment at the injury site and ^18^F-FDG uptake, which was further corroborated quantitatively (*r* = 0.81, *P* < 0.001) [80, 81]. Tawakol et al. confirmed a strong relationship between macrophage infiltration and ^18^F-FDG uptake in a model of nine male rabbits fed a high cholesterol diet and subject to balloon injury of the aortoiliac arterial segment [82]. Three to six months after injury* ex vivo* analysis showed an approximately 19-fold increase in ^18^F-FDG uptake in the upper abdominal aorta of the atherosclerotic group (108.9 ± 55.6% ID/g × 10^3^ versus 5.7 ± 1.2% ID/g × 10^3^; *P* < 0.001). Additionally histological assessment of macrophage density correlated highly with ^18^F-FDG uptake (*r* = 0.93,   *P* < 0.0001) with no association with wall thickness, plaque size, or density.

Early clinical studies by Rudd et al. showed increased ^18^F-FDG avidity in eight patients with carotid atherosclerosis (at least 70% internal carotid artery stenosis) and recent transient ischemic attacks. A 27% increase in ^18^F-FDG accumulation rate (plaque/integral plasma) in symptomatic lesions was demonstrated compared to the contralateral asymptomatic lesion [83]. More recent studies have confirmed increased ^18^F-FDG accumulation in rupture prone plaque of the coronary vasculature [84].

The Ruddy group investigated the feasibility of atherosclerotic plaque detection in the aorta in a retrospective analysis of 103 patients who had undergone ^18^F-FDG PET viability imaging due to severe coronary artery disease and systolic dysfunction. Ascending and descending thoracic aorta ^18^F-FDG avidity was graded based on peak and mean target-to-background ratio (TBR): grade 0 (<1), grade 1 (1.01–1.49), grade 2 (1.5–1.99), and grade 3 (>2.0). Increased FDG uptake was defined as grade 1–3 (TBR > 1), which represented 12% of the 103-patients [85].

^18^F-FDG is useful as a marker of vascular inflammation; however the metabolic processes of imminent plaque rupture involve a complex recruitment of multiple cell types responsive to various stimuli, which may not be adequately imaged by ^18^F-FDG PET. Furthermore, nonplaque myocardial uptake necessitating adequate extracardiac suppression can degrade image quality. PET radiotracers that more specifically target high-risk coronary disease would be more ideal for identifying atherosclerotic plaques prone to rupture.

### 4.2. Sodium ^18^F-Fluoride for Imaging Calcium Deposition

**Figure 9:**
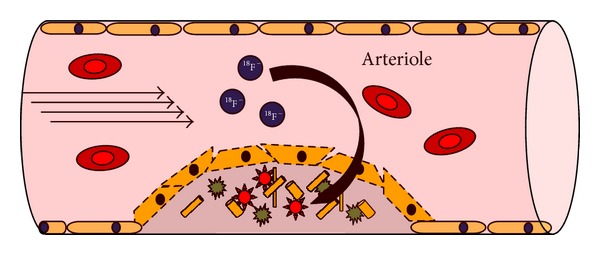
Schematic representation of the mechanism of ^18^F-NaF accumulation in an atherosclerotic plaque containing dysfunctional endothelium, microcalcification, and a large necrotic core (Table 8).

The ability to identify an atherosclerotic lesion at risk for rupture would enable earlier intervention and possibly improve outcomes. The implication is considerable since there currently are no other noninvasive methods to identify these vulnerable lesions. Joshi et al. in a recent analysis of 37 patients with myocardial infarction demonstrated the capacity of ^18^F-NaF to noninvasively localize within ruptured or coronary plaques at high risk of rupture [86] (Table 8). The mechanism of ^18^F-NaF uptake in ruptured or high-risk plaques reflects the end-stage of a controlled cellular inflammatory response from repeated insults (Figure 9). The authors postulate that osteoblastic metaplasia in response to inflammatory markers deposit hydroxyapatite in the calcified endothelium, particularly during the earliest and most active stages of mineralization. Hydroxyapatite nanocrystals nucleate, propagate, and mineralize the extracellular endothelial matrix which forms the site for ion-exchange between anionic ^18^F-fluoride and hydroxyl groups at the crystals surface. The ion-exchange process is most dependent on the area of the crystal surface, which is most prominent at plaque rupture or imminent rupture associated with inflammation and necrosis. The authors confirmed their hypothesis by demonstrating ^18^F-NaF uptake in regions of macrophage activation, necrosis, apoptosis, alkaline phosphatase, and osteocalcin staining.

**Figure 10:**
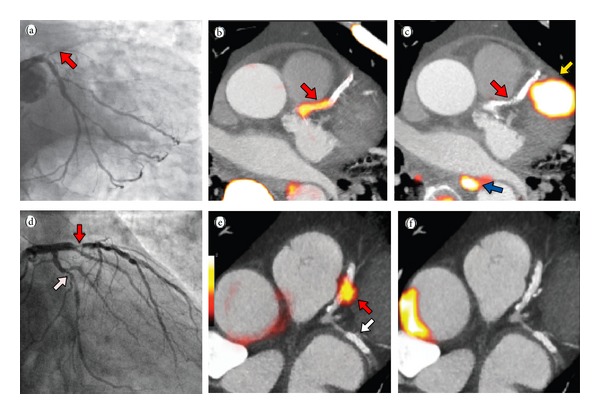
Acute STEMI patient with proximal LAD occlusion (red arrow) on angiography (a); subsequent ^18^F-NaF PET-CT imaging showing intense focal uptake in the culprit proximal LAD lesion (red arrow); (c) ^18^F-FDG PET-CT showing no uptake at the site of the culprit lesion (red arrow) with overlapping myocardial uptake of the culprit epicardial vessel; (d) Anterior NSTEMI patient with proximal LAD (red arrow) and LCx (white arrow) on angiography; focal intense uptake in only the culprit lesion (red arrow) on ^18^F-NaF PET-CT (e); and corollary ^18^F-FDG PET-CT showing no uptake in the culprit (LAD) or bystander (LCx) lesions (f) (reproduced with permission from Joshi et al. [86]).

In their cohort of 37 patients, all had acute myocardial infarction and all underwent both ^18^F-NaF and ^18^F-FDG ECG-gated PET images fused with CT coronary angiograms within a median of six days postinfarct (Figure 10). All subsequently underwent invasive coronary angiography with a median duration of 7 days between PET-CT and angiography. ^18^F-NaF activity in the coronary arteries of patients hospitalized for myocardial infarction was 34% higher than that in other epicardial vessels [maximum TBR = 1.66 {1.40–2.25} versus TBR = 1.24 {1.06–1.38}; TBR = tissue-to-background ratio]. To assess the capacity of ^18^F-FDG to image imminent atherosclerosis or plaque rupture, 28 patients with predefined suppression of myocardial uptake were imaged. Coronary ^18^F-FDG was essentially indistinguishable from heterogeneous myocardial uptake in 22 patients and increased uptake was observed in the culprit vessels of six. Overall TBR could not distinguish between culprit plaques and other coronary vasculature [TBR = 1.71 {IQR  1.40–2.13} versus TBR = 1.58 {1.28–2.01},  *P* = 0.34, mean difference of 0.09 (95% CI −0.07 to 0.24)]. In addition,* ex-vivo* ^18^F-NaF PET-CT was performed in nine specimens with localized uptake in the culprit lesion demonstrated.

For the first time, ruptured plaque or plaque at high risk of rupture can be identified noninvasively which as the authors postulate, carries the potential to alter management of stable and unstable coronary artery disease. Subsequent research will involve assessment of the capacity for ^18^F-NaF to risk stratify, monitor disease progression, guide therapy, and assist in the assessing treatment response in novel antiatherosclerotic therapy.

## 5. SPECT and PET Radiotracers Targeting Autonomic Dysfunction, Apoptosis, and Myocardial Infarct Repair

### 5.1. SPECT Imaging of Heart Failure with Norepinephrine Analogues (^123^I-MIBG)

The most extensively studied norepinephrine analogue for SPECT imaging of heart failure is ^123^I-*meta*-iodobenzyl guanidine (^123^I-MIBG)  (Table 9).^123^I-MIBG has similar storage, reuptake, and release in presynaptic sympathetic nerve terminals since norepinephrine does not undergo postsynaptic or presynaptic metabolism.

In the context of cardiac sympathetic innervation and heart failure, a hyperadrenergic state has been postulated leading to cascading release of norepinephrine from cardiac sympathetic postganglionic nerve terminals resulting in saturation of *β*-adrenergic receptors. Sustained saturation of *β*-adrenergic receptors leads to desensitization and eventual worsening of heart failure [87]. Furthermore, there is limited efficiency of norepinephrine reuptake by NET-1 leading to a sustained escape into the blood pool. This is the basis for treatment with *β*-blockers in patients with heart failure, which is known to reduce mortality. The ability to noninvasively assess myocardial adrenergic autonomic dysfunction has the potential to risk-stratify patients for therapeutic interventions.

**Figure 12:**
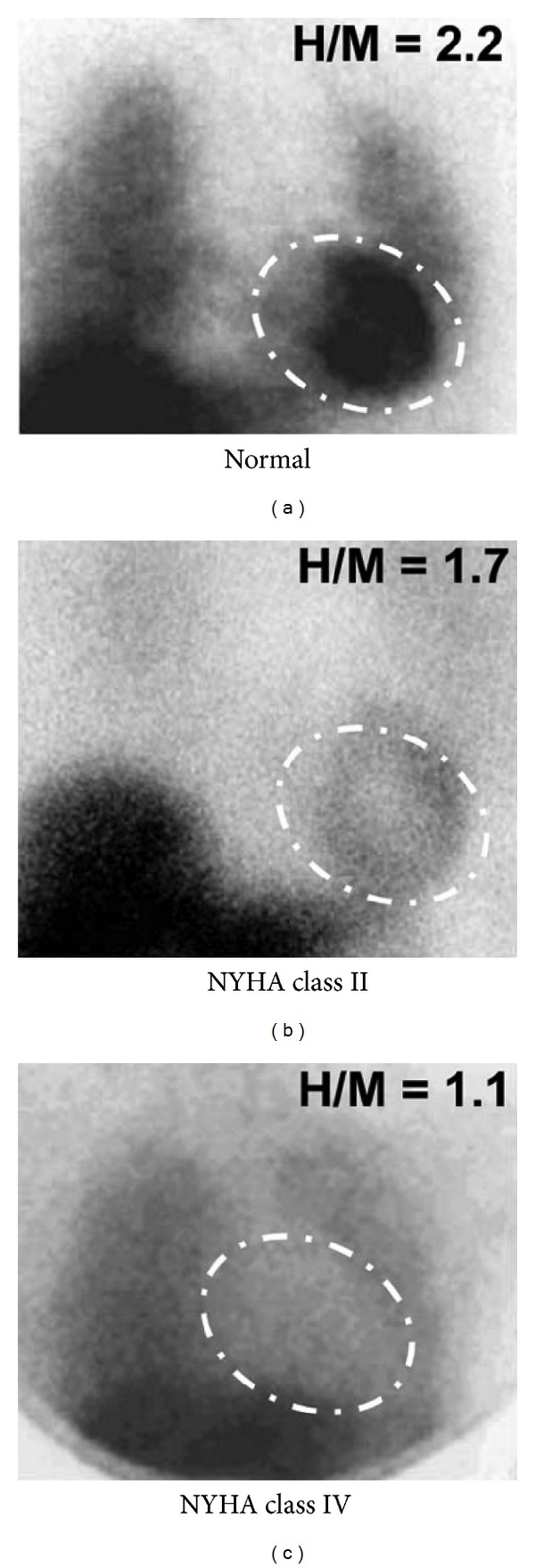
^123^I-MIBG planar images (anterior projection) in a normal patient and patients with CHF and worsening function by the New York Heart Association classification. Successively reduced uptake and heart-to-mediastinal ratios (H/M) with worsening NYHA class (reproduced with permission from Chen and Wu [88]).

Uptake of ^123^I-MIBG in heart failure patients reflects a balance between the capacity for ^123^I-MIBG storage in an intact presynaptic nerve terminal versus reuptake through NET-1 and release (a measure of sympathetic drive).^123^I-MIBG storage has been demonstrated semiquantitatively by a ratio of heart-to-mediastinal uptake in the first 10–20 minutes (early H/M) and sympathetic drive semiquantitatively as H/M uptake on late images (3-4 hours), which is essentially a measure of washout rate. A meta-analysis of ^123^I-MIBG imaging in congestive heart failure involving 18 studies demonstrated that either a low late H/M or high washout rate has poor prognostic implications including high mortality [89]. ADMIRE-HF trial [AdreView Myocardial Imaging for Risk Evaluation in Heart Failure; phase III with NYHA class II and III; LVEF <35% (AdreView = iobenguane ^123^I or ^123^I-MIBG)] demonstrated that a reduced H/M uptake on late imaging was associated with an increased risk of cardiac events (multivariate analysis)  (Figure 12) [90].

### 5.2. PET Imaging of Heart Failure with a Norepinephrine Analogue Radiotracer (^11^C-*Meta*-Hydroxyephedrine)

An interesting alternative to ^123^I-MIBG is the ephedrine analogue, ^11^C-*m*-HED, the characteristics of which are described in Table 10. Schwaiger et al. evaluated eleven patients (six normal volunteers and five patients with cardiac transplants) [91]. Normal volunteers showed heart/lung and heart/blood activity ratios of 4.2 and 5.0, respectively, 30 minutes after injection. The 2-minute cardiac retention of ^11^C-*m*-HED in the normal volunteers was 33 ± 16%, which remained relatively constant over 60 minutes. In contrast there was lower initial cardiac retention of ^11^C-*m*-HED in the cardiac transplant cohort (26 ± 8% at 2 minutes), which decreased at later time points (9 ± 3% at 60 minutes).  Ziegler et al. demonstrated that regional increases in ^11^C-*m*-HED uptake and retention in a cohort of 12 cardiac transplant patients coincided with functional sympathetic reinnervation of the left ventricle measured by a spectral analysis of heart rate variability [92] (Table 10).

In the context of congestive heart failure, Pietilä et al. demonstrated poor retention of ^11^C-*m*-HED (retention index = 0.184 ± 0.061; myocardial activity/integral time-activity-curve in plasma) in this cohort compared to healthy subjects (retention index = 0.283 ± 0.044).  Furthermore patients with a poor prognosis showed even lower retention (0.137 ± 0.041) [93, 94].

### 5.3. SPECT and PET Imaging of Apoptosis in Cardiovascular Diseases

**Figure 13:**
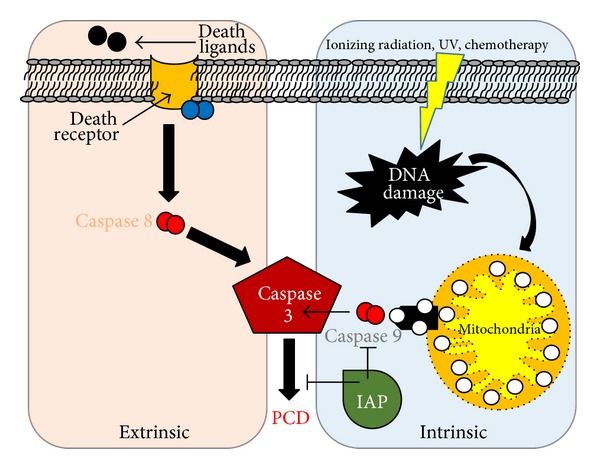
Apoptosis pathways. The intrinsic pathway (blue) in myocytes is mediated by mitochondria, initiated by release of cytochrome c (white spheres) into the cytosol or via extrinsic pathway (violet) through death receptors. Both processes result in caspase-3 activation that leads to a cleavage cascade that finally results in programmed cell death (PCD) {red spheres represent different subclasses of caspases. IAP = inhibitor-of-apoptosis (green); family of proteins that bind and inhibit caspases}.

Cardiac myocyte apoptosis plays a major role in atherosclerotic disease, myocardial ischemia and reperfusion injury, chronic heart failure, myocarditis, and cardiac allograft rejection. At the cellular level, apoptosis is a highly regulated energy dependent process that results in organized subcellular and cellular breakdown and does not involve inflammatory cell recruitment. Two pathways have evolved as targets for molecular imaging probes: (1) the extrinsic pathway that is facilitated by extracellular surface receptors and (2) the intrinsic pathway which involves the mitochondria and endoplasmic reticulum (Figure 13).

**Figure 14:**
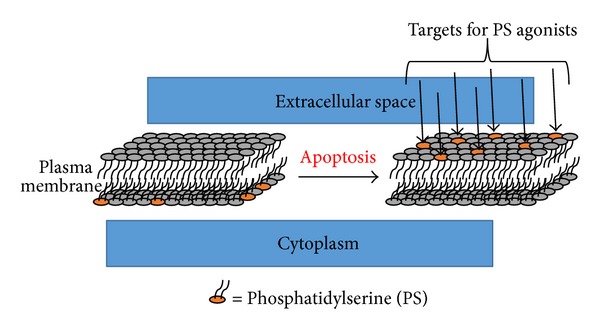
As part of the extrinsic pathway for apoptosis, phosphatidylserine (PS) normally on the inner leaflet of the plasma membrane is expressed on the outer leaflet [104]. Externalized PS is a target for annexin-5 through a calcium dependent binding mechanism [105].

Externalized phosphatidylserine on the apoptotic cell membrane has been studied as a target for the extrinsic pathway by multiple highly specific ligands, particularly the 37 kD protein annexin-V (Table 11). This protein has high affinity for phosphatidylserine and binds via a calcium dependent manner (Figure 14).^99m^Tc-radiolabelled annexin-V for SPECT has shown clinical promise in imaging carotid atherosclerosis [95], myocardial ischemia and reperfusion injury [96, 97], myocarditis [98], and in assessment of chemotherapeutic related cardiotoxicity [99]. A PET analogue of annexin-V radiolabelled with fluorine-18 was investigated by Yagle et al. [100]. The technically challenging radiochemistry precludes its routine clinical utility.

Kietselaer et al. in a study of 4 patients showed localization of ^99m^Tc-annexin-V preferentially in patients with transient ischemic attacks second to carotid artery atheroma. Furthermore they demonstrated selective uptake in more recent attacks, which is specific to macrophage recruitment [95]. A significant drawback of the annexin-V approach was demonstrated by the recent work of Wolters et al. who showed localization in nonapoptotic macrophages in addition to intraplaque hemorrhage [106].

Theoretically the most promising advance in molecular targeting of apoptosis is the rational design of probes with affinity for the apoptotic membrane imprint. The aforementioned apoptosis probes inherently suffer from the lack of true specificity for apoptosis since the processes they target overlap with necrosis or even viable ischemic cells in some instances. The apoptotic membrane imprint obviates this deficit since the processes targeted are a series of concerted events specific to the apoptotic pathway. This includes irreversible loss of plasma membrane potential, permanent acidification of the external plasma membrane/cytosol, and activation of the external membrane phospholipid scramblase system to attempt to preserve membrane integrity. Aposense Ltd. has created a series of novel small molecule PET radiotracers (^18^F-ML-10, ^18^F-ICMT-11, and ^18^F-BnTP) that selectively accumulate within the apoptosis-related complex (Table 11).

One of these complexes, ^18^F-ML-10, demonstrates selective uptake in apoptotic cells in concordance with the aforementioned hallmarks of the program cell death pathway, evidenced by signal loss at disruption of the cell membrane and thus is able to distinguish necrotic from apoptotic cells. After initial Phase I trials confirmed the safety, dosimetry, and biodistribution of this PET tracer, it has recently advanced to Phase IIa trials [107]. In a cohort of 10 patients with brain metastasis managed with radiation therapy, a correlation was demonstrated between ^18^F-ML-10 PET images 9-10 days after treatment and size changes detected by contrast-enhanced MR obtained 6–8 weeks after treatment [108]. With the goal of assessing this tracer in multiple tumour types, multicenter trials continue with this promising probe.

In contrast to its previously described exploration as a PET MPI agent, ^18^F-FBnTP is also under development as a “voltage sensitive probe” for apoptosis imaging through detection of membrane potential loss (Table 11).  One recently reported limitation is the susceptibility of this probe to premature efflux by multidrug resistant proteins thus producing a false positive test result for apoptosis [109].

### 5.4. PET Imaging of Myocardial Infarct Repair

Subcellular repair processes of cardiac myocytes after myocardial infarction are an interesting target for molecular imaging. Expression of *α*_*v*_ *β*_3_ integrin, an angiogenic cell membrane glycoprotein receptor on the surface of endothelial cells, is the target for a class of short-peptide-based radiopharmaceuticals based on the cyclic RGD peptide motif (Arg-Gly-Asp).  Under normal conditions this glycoprotein remains inactive. Upregulation is known to occur with a peak expression one-week after infarction as a marker of neovascularisation [110].

Laitinen et al. investigated ^18^F-galacto-cyclo(RGDfK) as a molecular target for *α*_*v*_ *β*_3_ integrin in a rat model of myocardial infarction [111]. At one week after MI, they demonstrated site-specific uptake of ^18^F-galacto-cyclo(RGDfK) in the infarct territory which had improved left ventricular remodelling at 12-weeks after injury. This important work demonstrated that early angiogenesis is an important prognostic factor for assessing left ventricular functional outcomes. However, due to the cumbersome synthesis of ^18^F-galacto-cyclo(RGDfK) and the requirement for an onsite or near-by cyclotron, this team began exploring alternative RGD derivatives using gallium-68 as the PET isotope with a variety of chelating agents.

Gallium-68 based PET radiotracers are attractive due to the efficient production of aqueous ^68^GaCl_3_ from ^68^Ge-^68^Ga generators allowing for easy coordination chemistry into a variety of chelates. Gallium-68 has favourable physical properties (*t*_1/2_ = 68 min, *E*_*β*max⁡_^+^ = 1.9 MeV). The prolonged half-life provides ample time for preparation of the radiotracer and the moderate positron energy (and range in soft tissue) allows high quality images. A series of gallium-68 labelled RGD analogues, specifically ^68^Ga-NODAGA-RGD (Table 12) and ^68^Ga-TRAP(RGD)_3_, were prepared and compared with ^18^F-galacto-RGD as *α*_*v*_ *β*_3_ integrin molecular probes in a rat model of myocardial infarction [112, 113]. One week after infarct created by left anterior descending (LAD) artery ligation, ^13^NH_3_ PET-CT images were obtained which confirmed relative hypoperfusion in the LAD territory. Subsequent injection with ^68^Ga-NODAGA-RGD, ^68^Ga-TRAP(RGD)_3_, or ^18^F-galacto-RGD demonstrated discordant uptake of the RGD derivatives in the infarct zone indicating early myocardial repair and angiogenesis. Immunohistochemical analysis confirmed increased density of the *β*_3_ integrin in the infarct zone. The tracer uptake ratios for remote (noninfarct tissue) versus infarct uptake were 4.7 ± 0.8, 5.2 ± 0.8, and 4.1 ± 0.7 for ^18^F-galacto-RGD, ^68^Ga-NODAGA-RGD, and ^68^Ga-TRAP(RGD)_3_, respectively.

## 6. Cardiac Molecular Imaging for MR and PET-MR

Highly paramagnetic lanthanides such as gadolinium (III) are under investigation for the preparation of a site-specific magnetic resonance (MR) contrast agents. Gd(III) in particular has seven unpaired electrons and long electronic relaxation times which are ideal for enhancing the relaxivity of nearby water molecules, the key feature in contrast enhancement for MRI. Gadolinium loosely coordinated to DOTA type chelates, for example, forms the basis for the majority of contrast agents in commercial use today. These agents, however, possess no intrinsic capacity for site specificity. Their mechanism of uptake reflects loose accumulation in high throughput vascular beds and is thus inadequate for assessing physiological processes.

Cardiac magnetic resonance (CMR) imaging continues to emerge as an important clinical tool and is becoming more widely available in cardiology imaging departments. The high spatial and temporal resolution, excellent soft tissue contrast, and nonionizing nature of image acquisition allow CMR to accurately assess both cardiac anatomy and function without exposure to radiation. CMR has the unique ability to assess features of coronary artery plaque by characterizing its water content, chemical composition, physical state, and molecular motion within the plaque [114–116]. Using late gadolinium enhancement, CMR is able to detect myocardial viability with similar specificity and slightly lower sensitivity than PET [117]. Molecular CMR imaging is performed using the paramagnetic gadolinium chelate covalently appended to a variety of target vectors including antibodies, peptides, or small molecules probes [118]. Other pathological processes including inflammation, atherosclerosis, and angiogenesis have been investigated with paramagnetic inorganic nanoparticles as MR contrast agents [118, 119]. Amirbekian et al. recently showed* in vivo* detection of macrophage activity in atherosclerotic plaques utilizing immunomicelles augmented with a gadolinium-diethylenetriaminepentaacetic acid (DTPA) coordination complex as a targeted molecular probe for the scavenger receptor on macrophages [120].

Hybrid PET-MR imaging systems initially focused on applications in the fields of neurology and pediatrics. Recently, some vendors are offering systems with simultaneous PET and MR acquisition, allowing heart rate and respiratory gated imaging, which is more adapted for cardiac applications. PET-MR combines the high soft-tissue resolution of MR with the sensitivity of PET, without any additional ionizing radiation. Unlike PET-CT, synergistic effects of the combination of PET and MR still need to be established.

Cardiac applications under investigation for PET-MR include the site-specific localization of culprit lesions to eventually guide intervention [113]. Preliminary data demonstrate that PET-MR could be used to quantify inflammatory response after infarction [121]. Other research interests for cardiac molecular imaging with PET/MR revolve around stem cell applications and neoangiogenesis [113].

## 7. Conclusions

Novel innovations in radiotracer design based on continuously emerging discoveries into the underlying subcellular mechanisms underpinning pathological processes form the basis for the expansive growth of cardiac molecular imaging. Technetium-99m radiopharmaceuticals continue as the workhorse for MPI; however novel tracers housing this unique radiometal are emerging with the potential for improved imaging characteristics and reduced radiation dosimetry. PET radiotracers have dramatically changed MPI showing improved overall diagnostic accuracy and prognosis. Other cardiac disease states such as heart failure, autonomic dysfunction, apoptosis, and neoangiogenesis have multiple site selective probes under active clinical investigation with promising implications for further insight and potential management considerations. Cardiac MR, while relatively new, has proven to be an interesting player in evaluating several of the aforementioned conditions devoid of radiation burden. Targeted molecular imaging for cardiac disease is a rich and diverse field with multiple promising avenues for ongoing research and development.

## Figures and Tables

**Figure 11:**
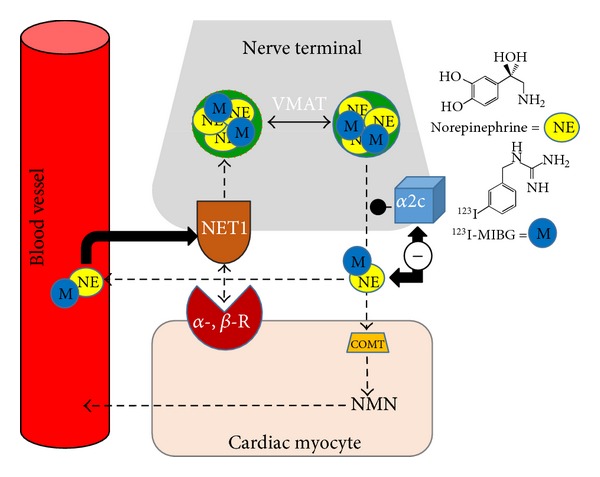
Mechanism of uptake of ^123^I-MIBG, an analogue of norepinephrine, in presynaptic sympathetic nerve terminals (adapted from Rogers et al.) [84] {catechol-*O*-methyl transferase = COMT; normetanephrine = NMN; alpha- and beta-adrenergic receptor subtypes = *α*-,  *β*-R and *α*2c; norepinephrine transporter 1 = NET1}.

**Table 1:** Commercial SPECT Radiotracers for Myocardial Perfusion Imaging.

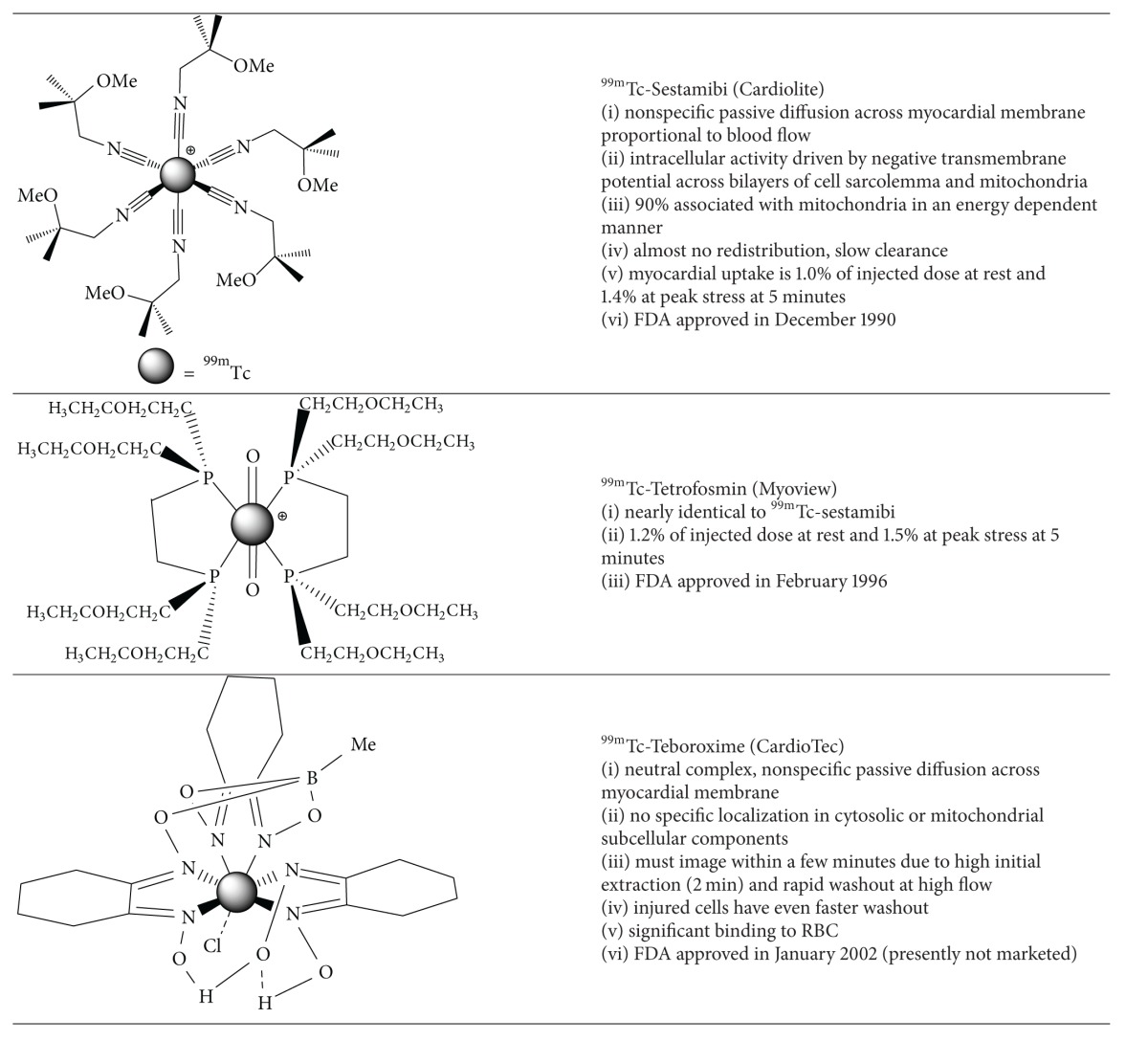

**Table 2:** Examples of Novel Technetium-99m SPECT Radiotracers for Myocardial Perfusion Imaging.

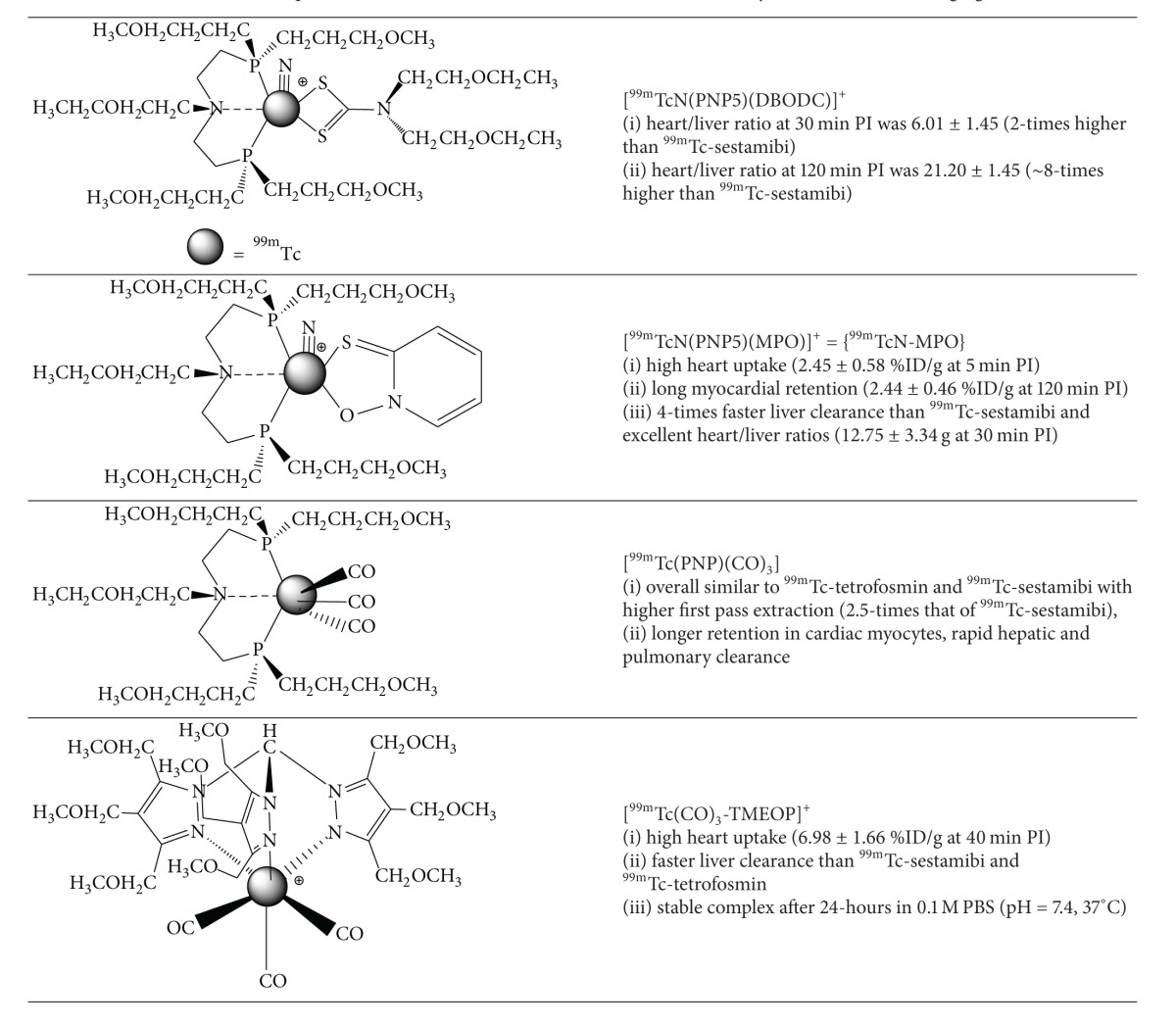

**Table 3:** Non-commercial/Novel SPECT Radiotracers for Myocardial Perfusion Imaging.

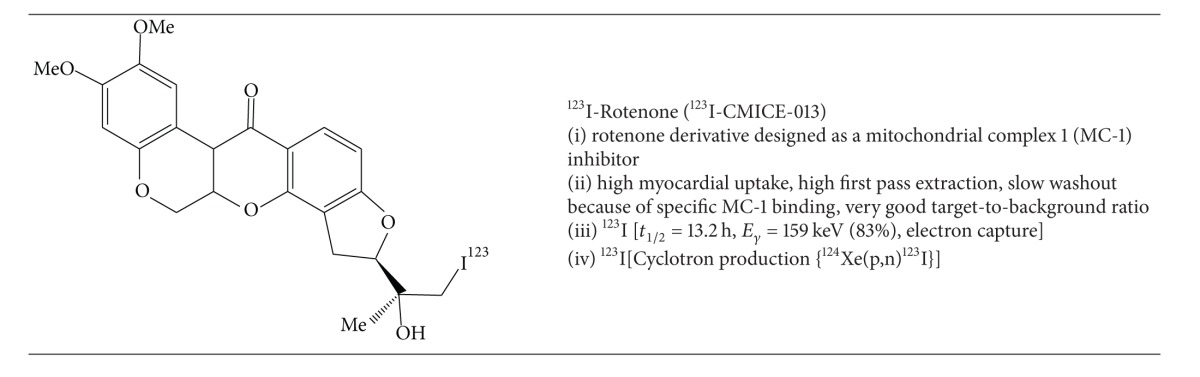

**Table 4:** Commercial PET Radiotracers for Myocardial Perfusion Imaging [51, 52].

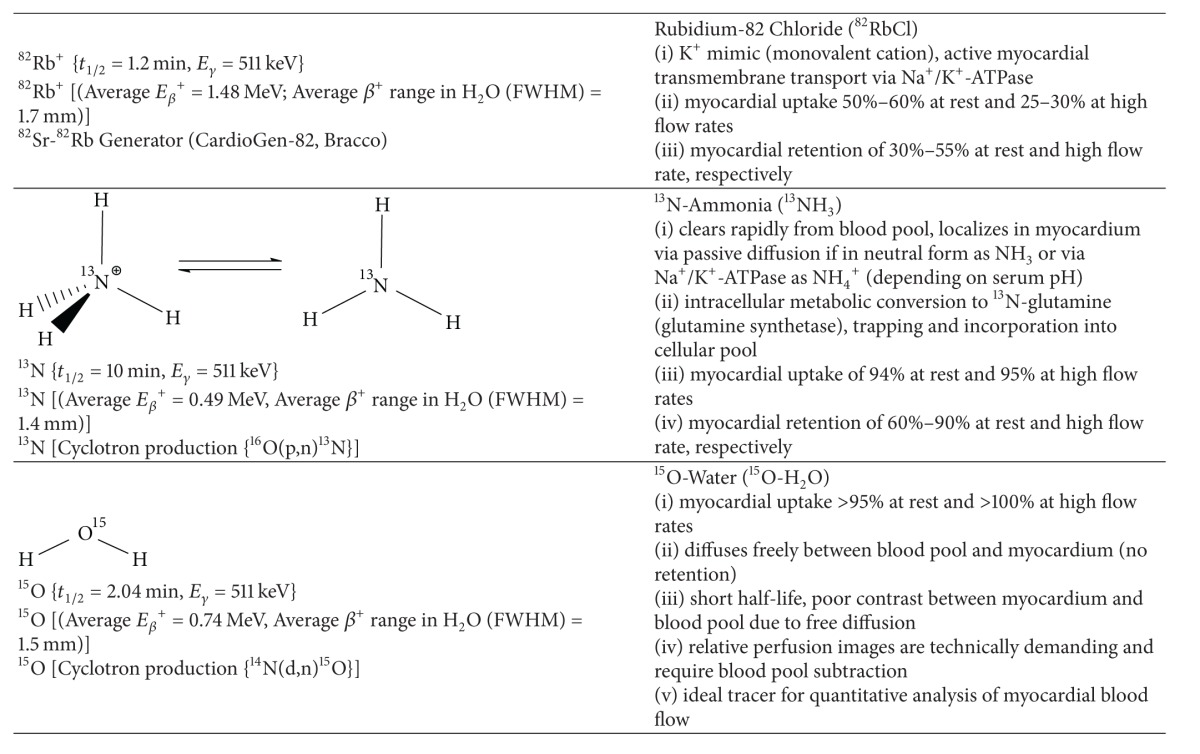

**Table 5:** Non-commercial/Novel PET Radiotracers for Myocardial Perfusion Imaging [53].

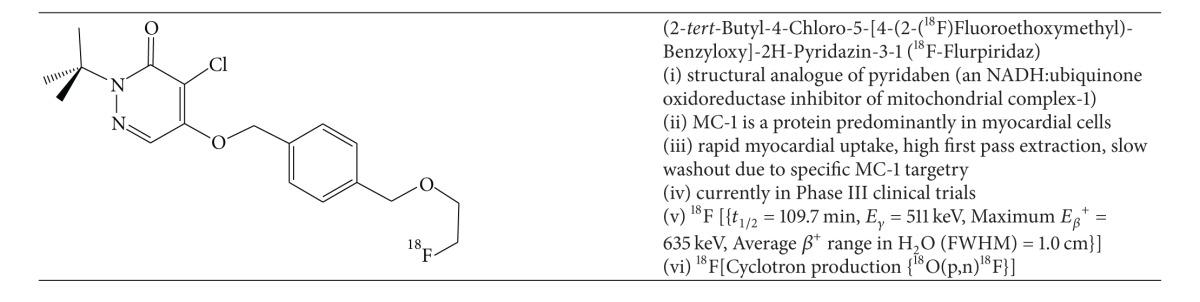

**Table 6:** PET Myocardial Metabolism Radiotracers for Evaluation of Viability or Inflammation.

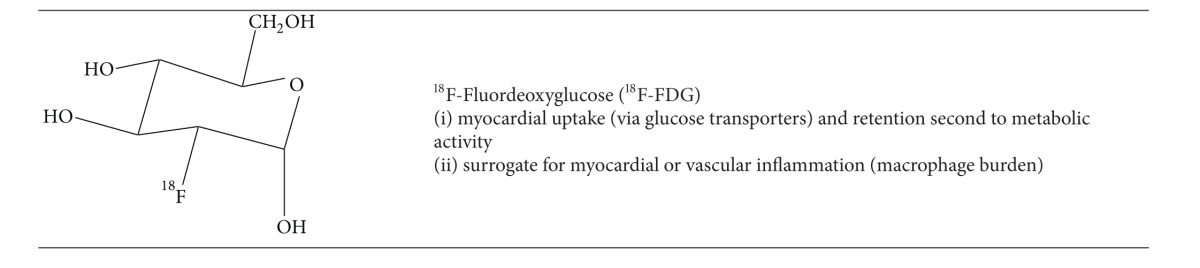

**Table 7:** SPECT Myocardial Metabolism Radiotracers.

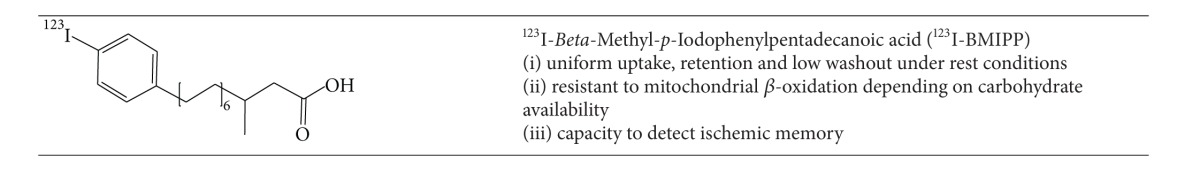

**Table 8:** PET Radiotracers for Imaging Atherosclerotic Plaques.

^ 18^F-NaF	Sodium ^18^F-Fluoride (^18^F-NaF) (i) capacity to identify ruptured or high-risk coronary atherosclerotic plaques (Figure 9) (ii) at risk plaques have positive remodelling, microCa^2+^ and large necrotic core (iii) fluoride ion (^18^F^−^) mimics hydroxyl ion and is deposited in nucleating hydroxyapatite crystals of calcified endothelium

**Table 9:** SPECT Radiotracers for Imaging Autonomic Dysfunction in Heart Failure.

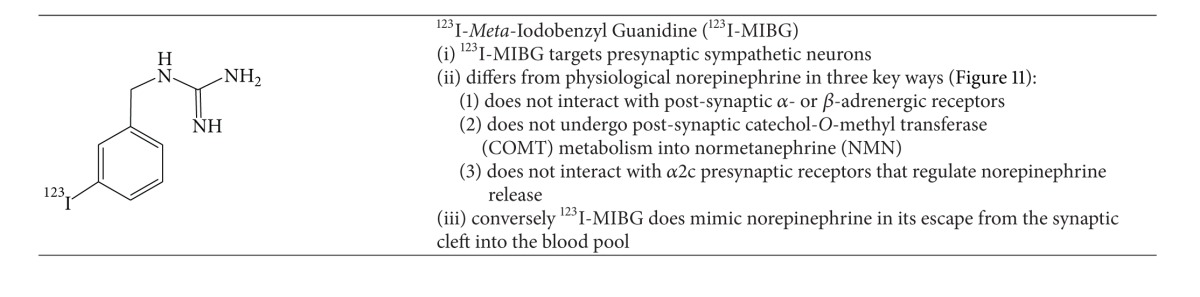

**Table 10:** PET Radiotracers for Imaging Autonomic Dysfunction in Heart Failure.

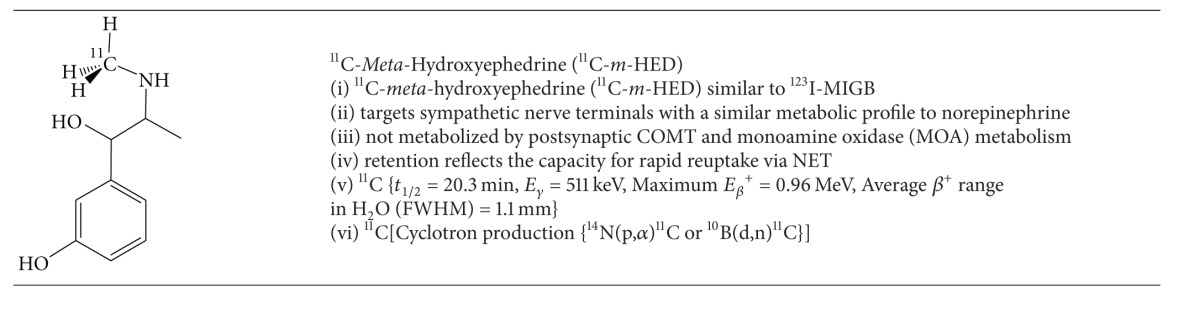

**Table 11:** SPECT and PET Radiopharmaceutical for Imaging Apoptosis.

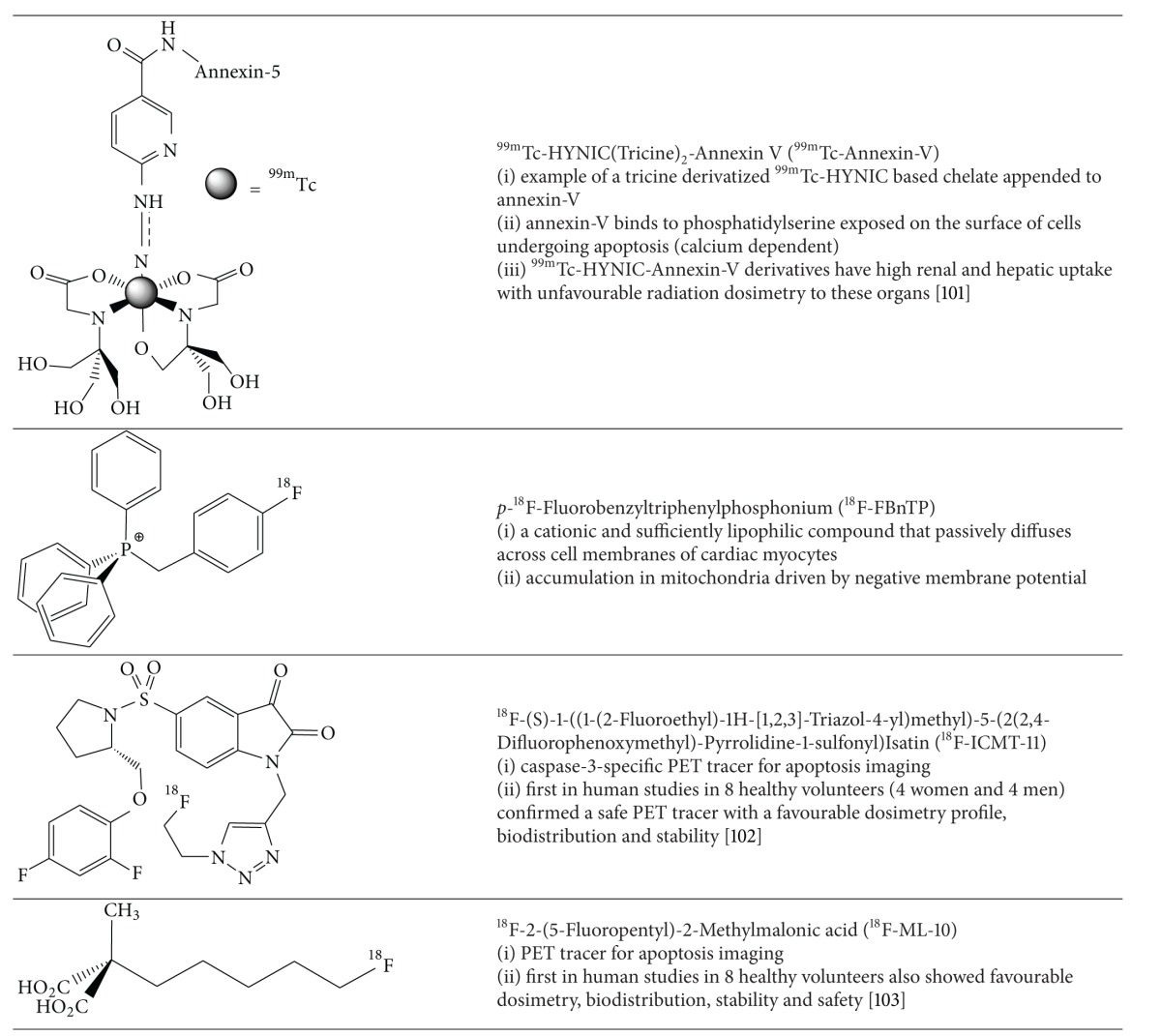

**Table 12:** PET Radiopharmaceutical for Imaging Post-infarct Repair.